# High ABCG4 Expression Is Associated with Poor Prognosis in Non-Small-Cell Lung Cancer Patients Treated with Cisplatin-Based Chemotherapy

**DOI:** 10.1371/journal.pone.0135576

**Published:** 2015-08-13

**Authors:** Guang Yang, Xue-Jiao Wang, Li-Jun Huang, Yong-An Zhou, Feng Tian, Jin-Bo Zhao, Peng Chen, Bo-Ya Liu, Miao-Miao Wen, Xiao-Fei Li, Zhi-Pei Zhang

**Affiliations:** Department of Thoracic Surgery, Tangdu Hospital, the Fourth Military Medical University, Xi’an, 710038, China; Hungarian Academy of Sciences, HUNGARY

## Abstract

ATP-binding cassette (ABC) transporters are associated with poor response to chemotherapy, and confer a poor prognosis in various malignancies. However, the association between the expression of the ABC sub-family G member 4 (ABCG4) and prognosis in patients with non-small-cell lung cancer (NSCLC) remains unclear. NSCLC tissue samples (n = 140) and normal lung tissue samples (n = 90) were resected from patients with stage II to IV NSCLC between May 2004 and May 2009. ABCG4 mRNA and protein expressions were detected by RT-PCR, western blot, and immunohistochemistry. Patients received four cycles of cisplatin-based post-surgery chemotherapy and were followed up until May 31^st^, 2014. ABCG4 positivity rate was higher in NSCLC than in normal lung tissues (48.6% *vs*. 0%, *P*<0.001) and ABCG4 expression was significantly associated with poor differentiation, higher tumor node metastasis (TNM) stage, and adenocarcinoma histological type (all *P*<0.001). Univariate (HR = 2.284, 95%CI: 1.570–3.324, *P*<0.001) and multivariate (HR = 2.236, 95%CI: 1.505–3.321, *P*<0.001) analyses showed that ABCG4 expression was an independent factor associated with a poor prognosis in NSCLC. Patients with ABCG4-positive NSCLC had shorter median survival than ABCG4-negative NSCLC (20.1 *vs*. 43.2 months, *P*<0.001). The prognostic significance of ABCG4 expression was apparent in stages III and IV NSCLC. In conclusion, high ABCG4 expression was associated with a poor prognosis in patients with NSCLC treated with cisplatin-based chemotherapy.

## Introduction

About 1.5 million new cases of lung cancer are diagnosed annually worldwide, and about 85% of them are non-small-cell lung cancers (NSCLCs) [1]. In 2012, about 58% of cases of NSCLCs occurred in the less developed countries [2]. Surgery is considered to be the best treatment option, but resection is only potentially curative for 20–25% of tumors [1]. Most patients will present with an incurable disease and face a 5-year relative survival rate of <17% with current standard therapies [3]. Postoperative chemotherapy, with or without radiotherapy, improves survival by 4% in terms of absolute 5-year survival, as demonstrated by a meta-analysis [4].

Currently, cisplatin-based combination chemotherapy regimens are the most effective first-line therapies for the treatment of NSCLC, according to The National Comprehensive Cancer Network (NCCN) guidelines (version 4.2014) [5]. However, the efficacy of chemotherapy is limited, with response rates (RRs) of 20–35%, progression-free survival (PFS) of 3.1–5.5 months, and overall survival (OS) 7.4–11.3 months [6,7]. Accordingly, improving outcomes after chemotherapy has been one of the most challenging issues in the successful treatment of lung cancer [8]. This is primarily attributed to drug resistance, which occurs in virtually all cancers and is a serious impediment to the success of chemotherapy [6,7]. It is increasingly recognized that primary and secondary drug resistances developing in cancer cells are the most fundamental reason underlying chemotherapy failure [6,7].

Drug resistance is usually the result of the over-expression of ATP-binding cassette (ABC) transporters [9,10]. ABC transporter proteins are optimal candidates as biomarkers for the prediction of chemotherapy resistance and prognosis after chemotherapy [11,12]. ABC transporters are transmembrane proteins that facilitate translocation of heterogeneous substances across cellular membranes by utilizing the energy of ATP hydrolysis [13]. To date, at least 48 human ABC transporter genes have been identified, and they have been divided into seven subfamilies (ABCA through ABCG) [14]. Among these, ABCA2, ABCB1 (also known as P-glycoprotein or multidrug resistance 1 (MDR1)), ABCB4, ABCB11, ABCC1-6, ABCC10, ABCC11, and ABCG2 (also known as BCRP or MXR) have all been shown to be associated with the efflux and transportation of cytotoxic drugs (structurally diverse chemotherapeutic agents and their metabolites) from lung cancer cells, thus reducing intracellular drug concentrations [15]. ABCA1 has also been identified as mediating drug resistance in lung cancer and drug-resistant lung cancer cell lines [16]. Currently, P-gp (ABCB1), MRP1 (ABCC1), and ABCG2 are considered to be the main drug transporters for their roles in multi-drug resistance observed in many tumors and cell lines [17]. Although discovery and use of inhibitors for ABC transporters have been studied over the past years, these inhibitors were not completely successful in overcoming drug resistance [18]. As a result, identification of new ABC drug transporter proteins and investigation of their mechanisms and inhibitors are highly warranted and extremely important to evaluate new targets for overcoming drug resistance.

Several studies have demonstrated that the members of the ABCG (or White) subfamily are associated with cellular lipid transport and drug resistance [19–21]. ABCG5 and ABCG8 are highly expressed in epithelial cells of the intestine and likely act as a heterodimer; they have been associated with the efflux of plant sterols and cholesterol into bile [22,23]. Over-expression of ABCG2 leads to resistance of various cancer cell lines to anti-tumor drugs [24]. ABCG4 is the fourth member of the "G" family, and is located on chromosome 11q23.3. This gene encodes a 3874-bp transcript, and its expression is predominantly intracellular [25], but the exact intracellular localization is yet to be shown [26]. Indeed, the different ABC transporters may be localized in the cell membrane, on mitochondria, on the Golgi apparatus, on the endoplasmic reticulum, or in the nuclear membrane, where they participate in different cellular processes [26]. Moreover, according to previous studies [27–30], ABCG4 expression in NSCLC remains unclear. Previous studies have revealed that ABCG1 and ABCG4 are responsible for the transportation of cholesterol onto high density lipoprotein (HDL) particles [31,32], and over-expression of ABCG4 also confers resistance to alkyl-phospholipid analogues (miltefosine, edelfosine, and perifosine) in *Leishmania* [33]. However, whether ABCG4 plays a significant role in prognosis after chemotherapy, and whether it has potential function of mediating drug resistance in lung cancer also remain unclear. Therefore, the present study focused on understanding the expression and post-chemotherapy prognostic value of ABCG4 in NSCLC.

In this retrospective study, we investigated the expression of ABCG4 in NSCLC. We also evaluated the value of ABCG4 protein for predicting survival in NSCLC patients treated with cisplatin-based combination chemotherapy.

## Materials and Methods

### Patients and samples

A total of 140 NSCLC tissue samples (74 cases of lung squamous cell carcinoma and 66 cases of lung adenocarcinoma) and 90 normal lung tissue samples (located >5 cm from the primary tumors) obtained by surgical resection at the Department of Thoracic Surgery, Tang Du Hospital Affiliated to the Fourth Military Medical University (Xi'an, China) between May 2004 and May 2009, were investigated in the present study. Tumor and normal lung tissues were harvested using sterile surgical instruments.

Patients were aged 45–76 years (mean ± SD: 60.76 ± 6.88 years). All samples were reviewed by pathologists, and histological classification of tumors was performed according to the World Health Organization criteria. Tumors were classified as stage II (n = 42), III (n = 56), or IV (n = 42) according to the NCCN guidelines (Version 4.2014) [5]. Clinicopathological characteristics of all patients were recorded. No patient had received chemotherapy, radiotherapy, biotherapy, or any other operation before lung cancer surgery. All patients were treated with cisplatin-based combination chemotherapeutic regimens considered as standard postoperative regimens for NSCLC [5]. All patients received four cycles of cisplatin-based combination chemotherapeutic regimens (cisplatin 75 mg/m^2^ day 1+gemcitabine 1250 mg/m^2^ days 1, 8, every 21 days/pemetrexed 500 mg/m^2^ day 1 every 21 days/docetaxel 75 mg/m^2^ day 1 every 21 days). Follow-up was censored on May 31^st^, 2014, with a total follow-up period of 60 months. The day when chemotherapy started was considered as the starting point for estimating postoperative survival.

All specimens were divided in two parts: the first part was placed in a 0.1% diethylpyrocarbonate (DEPC) water-treated freezing tube and stored at -80°C; the second part was fixed in 10% neutral buffered formalin and then dehydrated for paraffin embedding.

The study protocol was approved by the Regional Ethics Committee for Clinical Research of the Fourth Military Medical University. All patients provided written informed consent for the use of their medical records and tissue specimens for research purposes.

### Reverse transcriptase-polymerase chain reaction (RT-PCR)

Frozen tissue samples (100 mg) were harvested and ground into a fine powder. A549 cells were stably transfected with the pcDNA3.1 vector containing ABCG4 (Takara Bio, Otsu, Japan) using Lipofectamine 2000 (Invitrogen Inc., Carlsbad, CA, USA), according to the manufacturer’s protocol. Cells were seeded in 6-well plates, and DNA plasmids and transfection reagent were added 24 h later. Transfected A549 cells were cultured in the presence of G418 (400 mg/ml; Life Technologies Co., Grand Island, NY, USA). G418-resistant cells (A549-ABCG4) were picked for RT-PCR analysis.

RNA extraction was carried out using the Total RNA Kit I (Omega, Norcross, GA, USA). The concentration and purity of RNA samples were determined using absorbance at 260 and 280 nm (A260/280) by spectrophotometry (Beckman DU800, Beckman Coulter Inc., Brea, CA, USA).

cDNA synthesis was performed using a reverse transcription kit (Thermo Fisher Scientific, Waltham, MA, USA) using the Random Hexamer Primer, and RevertAid M-MuLV Reverse Transcriptase. PCR amplification was performed using 2×Taq Mastermix (CWBio, Beijing, China). Initial denaturation was conducted at 95°C for 5 min, followed by 30 cycles of denaturation at 95°C for 35 s, annealing at 51°C for 35 s, and extension at 72°C for 35 s, with a final extension at 72°C for 5 min (Table 1). PCR products were analyzed on 2% agarose gels using a gel imager (Alpha Innotech, Santa Clara, CA, USA).

**Table 1:** Primers and Conditions for RT-PCR.

Target gene	Primer sequences	Productlength (bp)	Annealingtemperature (°C)	Number of cycles
**ABCG4**	F: 5’-CTGAGTGAGAAGCAGGAGGT-3’	383	51	30
	R: 5’-AAGCCGAGTCCCTTTAGAT-3’			
**β-actin**	F: 5’-GAGCTACGAGCTGCCTGACG-3’	416	51	30
	R: 5’-CCTAGAAGCATTTGCGGTGG-3’			

*Note*: ABCG4: ATP-binding cassette sub-family G member 4; F: Forward; R: reverse; bp: base pairs.

### Western blot analysis

Frozen tissue samples (100 mg) were cut into small pieces and homogenized in a radioimmunoprecipitation assay (RIPA) buffer [1×PBS, 0.1% sodium dodecyl sulfate (SDS), 1% Nonidet P-40, 0.5% sodium deoxycholate, 10 μg/ml leupeptin, 100 μg/ml p-aminophenylmethylsulfonyl fluoride (p-APMSF) and 10 μg/ml aprotinin] on ice for 1 h, and centrifuged at 14,000 ×*g* for 30 min. A549-ABCG4 cells were lysed with the RIPA buffer on ice for 1 h and centrifuged at 14,000 ×g for 30 min. The supernatant was then harvested and the protein concentration was determined using the bicinchoninic acid (BCA) protein assay. Protein lysates (50 μg) were separated by 12% SDS-polyacrylamide gel electrophoresis (PAGE) and transferred onto polyvinylidene fluoride (PVDF) membranes at 2.5 mA/cm^3^ for 30 min. Membranes were blocked with 5% skimmed milk powder for 1 h at room temperature and incubated with the primary antibodies (anti-ABCG4 rabbit polyclonal antibody, 1:1000, Proteintech, Chicago, IL, USA; and anti-β-actin rabbit monoclonal antibody, 1:1000, CW Bio, Beijing, China) at 4°C, overnight. Membranes were then washed with TBST buffer (10 mM Tris-HCl, pH 8.0, 150 mM NaCl and 0.1% Tween-20), and subsequently incubated with the secondary antibody (goat anti-rabbit antibodies, 1:5000, CW Bio, Beijing, China) at 37°C for 1 h, followed by color development using the Milipore chromogenic kit for western blots (Billerica, MA, USA) and observation under a chemiluminescence analyzer (Bio-Rad, Hercules, CA, USA).

### Immunohistochemistry

Paraffin-embedded tissues were cut into 3-5-μm sections for dewaxing and rehydration. Immunohistochemistry was performed after microwave heating-based antigen retrieval in 0.1 M citrate buffer (pH = 6.0) followed by incubation with 3% H_2_O_2_ at room temperature for 30 min, 10% goat serum at room temperature for 30 min, primary antibody (anti-ABCG4 rabbit polyclonal antibody, 1:40, Proteintech, Chicago, IL, USA) overnight at 4°C, biotin-labeled secondary antibody (CW Bio, Beijing, China) at room temperature for 30 min, and freshly prepared 3,3'-diaminobenzidine (DAB) for color development for 5 min. Sections were then thoroughly rinsed with water, re-stained with hematoxylin, dehydrated, cleared in xylene and mounted with a coverslip. The specificity of the anti-ABCG4 antibody was tested using ABCG4-blocking peptides (1:200; sc-34874; Santa-Cruz Biotechnology, Santa Cruz, CA, USA). The blocking peptides were added to the slides and incubated overnight at 4°C. Then, the primary antibody was added and revealed as described above.

Specimens were classified as "positive" when the cellular membrane and/or cytoplasm were stained brown. Five high-power fields (400×) were selected at random, and each slide was evaluated independently by two experienced pathologists blinded to the clinical data. Phosphate-buffered saline (PBS), instead of the primary antibody, was used as a blank control.

Specimens were scored according to the staining intensity and the percentage of positive cells. The results were scored based on the following criteria: a) the percentage of positive cells (≤5%: 0; 6–25%: 1; 26–50%: 2; 51–75%: 3; and >75%: 4); b) the staining intensity (no color: 0; yellow: 1; brown: 2; and tan: 3); and c) the two grades were multiplied together and specimens were assigned to one of 4 levels: 0: negative (-); 1–4: weakly positive (+); 5–8: moderately positive (++); 9–12: strongly positive (+++) [34]. For multivariate analysis and comparison with the survival curves,–was defined as negative, and +, ++ and +++ were all defined as positive.

### Statistical analysis

Statistical analyses were performed using SPSS 18.0 (IBM, Armonk, NY, USA). Associations between immunohistochemical expression and clinical variables were evaluated by Mann-Whitney U or Kruskal-Wallis H test, as appropriate. Overall survival was measured from the start of chemotherapy to the date of death from any cause or the date the patient was last deemed to be alive. Survival curves were estimated by the Kaplan-Meier method, and compared using the log-rank test. The Cox proportional hazards model was used for univariate and multivariate analyses. Correlations were assessed using the Spearman rank correlation analysis. The rank correlation coefficients were expressed as *r*_s_. 0.0≤*r*_*s*_<0.1 was regarded as uncorrelated; 0.1≤*r*_*s*_<0.3 was regarded as weakly correlated; 0.3≤*r*_*s*_≤0.5 was regarded as moderately correlated; *r*_*s*_>0.5 was regarded as a strongly correlated. Two-sided *P*-values<0.05 were considered to be statistically significant.

## Results

### ABCG4 mRNA and protein expressions in NSCLC and normal lung tissues

**Fig 1:**
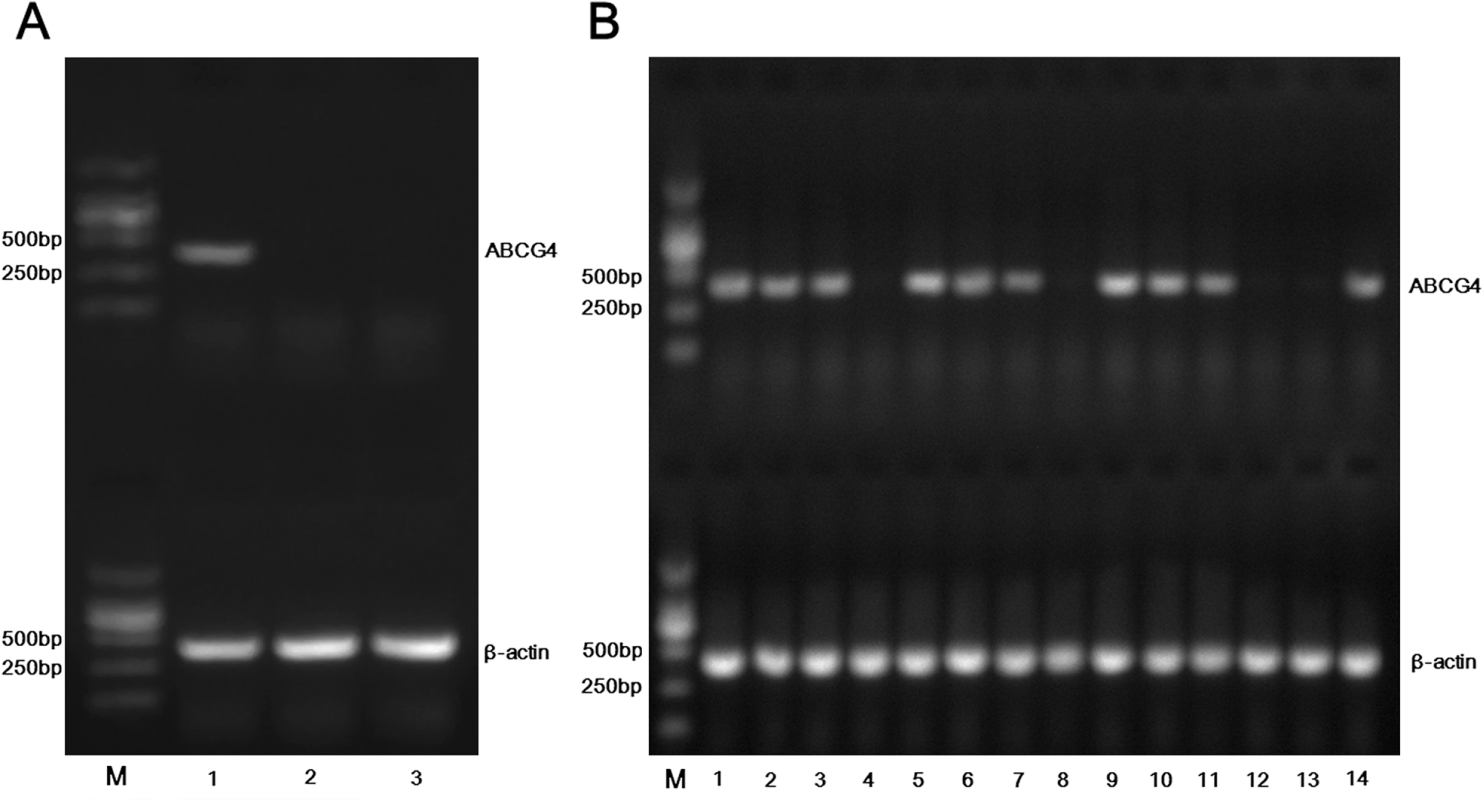
ATP-binding cassette sub-family G member 4 (ABCG4) mRNA expression was detected in non-small-cell lung cancer (NSCLC) (n = 14) and normal lung (n = 2) tissues by reverse transcription-polymerase chain reaction (RT-PCR). β-actin was used as an internal reference. M: marker. (**A)** Lane 1: positive control (A549-ABCG4). Lanes 2–3: normal lung tissues (NORM) (n = 2); **(B)** NSCLC tissues (NSCLC) (n = 14, lanes 1–14).

To investigate ABCG4 mRNA expression in NSCLC and normal lung tissue samples, RT-PCR was performed on mRNA isolated from the NSCLC tissues (n = 14) and normal lung tissues (n = 2). Detection of a 383-bp PCR product corresponding to ABCG4 mRNA was observed in NSCLC tissues (Fig 1B), while no such band was observed in normal lung tissues (Fig 1A). Amplification of β-actin (416 bp) was clearly observed in both tissue types (Fig 1).

**Fig 2:**
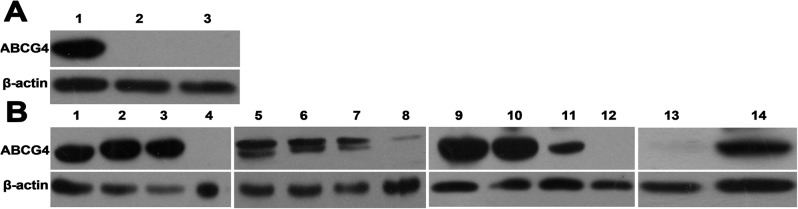
ABCG4 protein expression was detected in NSCLC (n = 14) and normal lung (n = 2) tissues by Western blot, using the same set of samples as in Fig 1. Full blots were shown in the S1 Fig β-actin (43 kDa) was used as an internal reference. (**A)** Lane 1: positive control (A549-ABCG4). Lanes 2–3: normal lung tissues (NORM) (n = 2); **(B)** NSCLC tissues (NSCLC) (n = 14, lanes 1–14).

To investigate ABCG4 protein expression in NSCLC and normal lung tissues, western blot analysis was performed in the set of samples used for RT-PCR. ABCG4 protein expression (60 kDa) was observed in NSCLC tissues (Fig 2B and S1 Fig), but not in normal lung tissues (Fig 2A). β-actin (43 kDa) was used as a normalization control and was observed in both tissue types (Fig 2 and S1 Fig).

These results indicated that ABCG4 mRNA and protein expressions were obviously different between NSCLC and normal lung tissues.

### Expression of ABCG4 protein in NSCLC and normal lung tissues by immunohistochemistry

**Fig 3:**
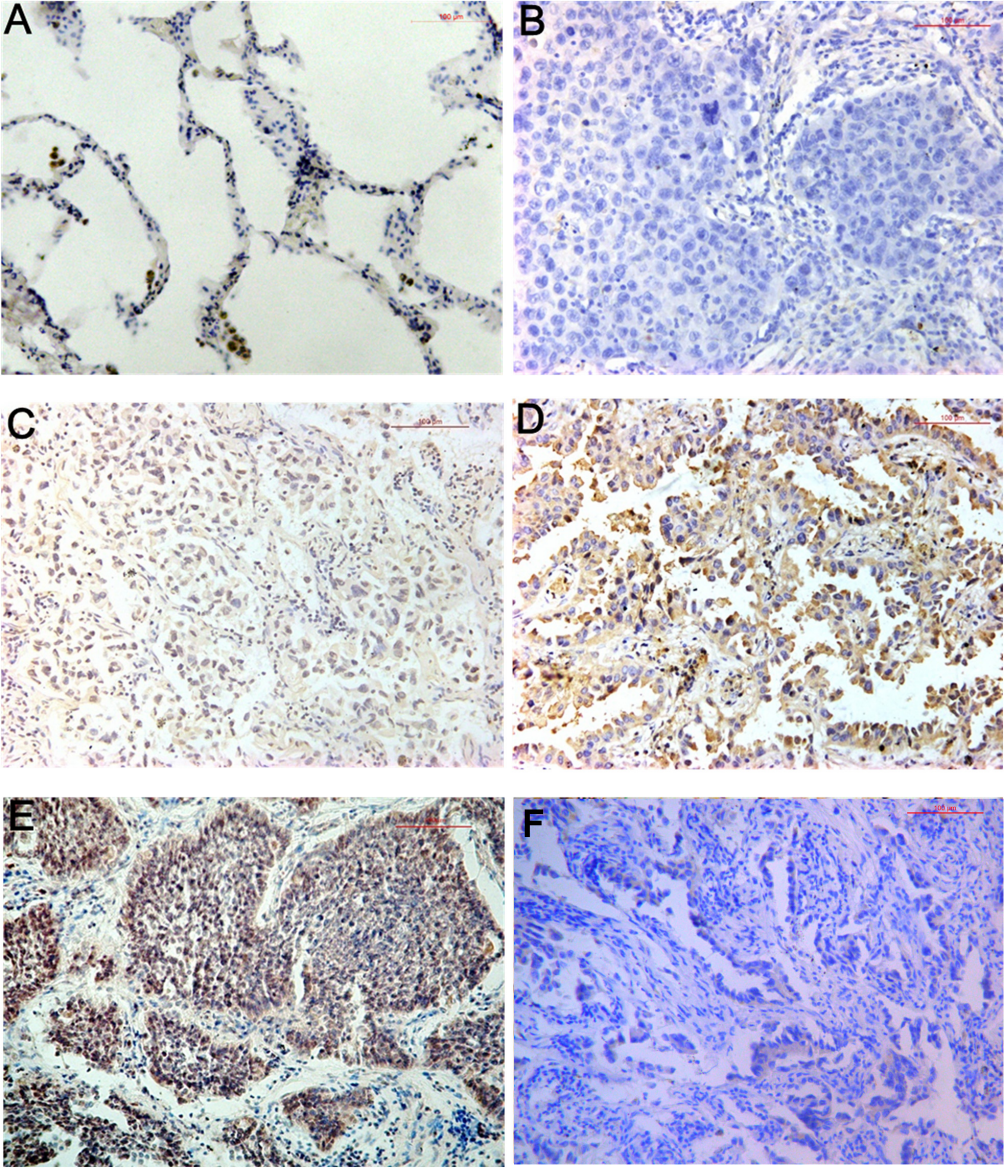
ABCG4 protein expression in NSCLC and normal lung tissues was detected by immunohistochemistry. (A) Normal lung tissue; (B) Negative (-) staining of ABCG4 in NSCLC tissue; (C) Weakly positive (+) staining of ABCG4 in NSCLC tissue; (D) Moderately positive (++) staining of ABCG4 in NSCLC tissue; (E) Strongly positive (+++) staining of ABCG4 in NSCLC tissue; (F) Immunohistochemistry showing ABCG4-negative staining in NSCLC tissues (++) using ABCG4 blocking peptide. Magnification: 200×.

To further investigate the expression of ABCG4 protein in NSCLC, immunohistochemistry was performed on NSCLC (n = 140) and normal lung tissues samples (n = 90) (Fig 3). No ABCG4 expression was seen in the normal tissue samples (Fig 3A). Some of the NSCLC samples also showed no expression (-) (Fig 3B), while other samples were weakly positive (+) (Fig 3C), moderately positive (++) (Fig 3D), or strongly positive for ABCG4 (+++) (Fig 3E). ABCG4-negative staining was showed in the NSCLC tissue samples (++) using ABCG4 blocking peptide (Fig 3F). Based on immunohistochemistry, ABCG4 expression was detected in 48.6% of NSCLC cases (68/140, 40 +, 23 ++, and 5 +++), and was not detected in any of the 90 normal lung tissue samples (*P*<0.001) (Table 2).

**Table 2:** ABCG4 Expression in Non-small-cell Lung Cancer (NSCLC) and Normal Lung Tissues Determined by Immunohistochemistry.

Tissues	N	ABCG4 expression					
		-	+	++	+++	PR (%)	*P*
**NSCLC**	140	72	40	23	5	48.6	<0.001
**Normal lung**	90	90	0	0	0	0.0	

*Note*: ABCG4: ATP-binding cassette sub-family G member 4; PR: positive ratio.

–no expression, + weakly positive, ++ moderately positive, and +++ strongly positive for ABCG4 expression.

These results suggested that ABCG4 was expressed in NSCLC tissues, and that it was not expressed in normal lung tissues.

### Association between ABCG4 expression and clinicopathological characteristics in patients with NSCLC

Having observed that ABCG4 was overexpressed in almost half of the NSCLC tissue samples, the association between ABCG4 expression and the characteristics of the patients were investigated. There was no significant correlation with gender, age, or smoking habits (all *P*>0.05). However, high ABCG4 expression was significantly correlated with lung adenocarcinoma (53.0%) rather than lung squamous cell carcinoma (44.6%) (*P*<0.001), in poorly (55.2%) rather than well/moderately (42.5%) differentiated NSCLC (*P* = 0.008), and with increasing tumor node metastasis (TNM) stage II, III and IV (31.0% *vs*. 53.6% *vs*. 59.5%, respectively) (*P*<0.001) (Table 3). Moreover, ABCG4 expression levels were weakly positively correlated with the increasing TNM stage (*r*_*s*_ = 0.210, *P* = 0.013) (data not shown).

**Table 3:** Relationship between ABCG4 Expression and Clinicopathological Characteristics in NSCLC Patients.

Variables		N	ABCG4 expression				PR (%)	*P*
			-	+	++	+++		
**Gender**								
	**Male**	66	35	22	6	3	47.0	0.179
	**Female**	74	37	18	17	2	50.0	
**Age (years)**								
	**≥60**	63	33	21	5	4	47.6	0.119
	**<60**	77	39	19	18	1	49.4	
**Smoking (packs per year)**								
	**≥20**	71	38	18	10	5	46.5	0.084
	**<20**	69	34	22	13	0	50.7	
**Differentiation**								
	**Poor**	67	30	23	13	1	55.2	0.008
	**Moderate/well**	73	42	17	10	4	42.5	
**TNM stage**								
	**II**	42	29	8	5	0	31.0	<0.001
	**III**	56	26	16	12	2	53.6	
	**IV**	42	17	16	6	3	59.5	
**Histology**								
	**Adenocarcinoma**	66	31	18	14	3	53.0	<0.001
	**Squamous cell carcinoma**	74	41	22	9	2	44.6	

*Note*: TNM: tumor-node-metastasis; PR: positive ratio.

These data suggested that ABCG4 expression was associated with tumor differentiation, TNM stage and histological type, and that ABCG4 expression levels were positively associated with TNM stage.

### Association between ABCG4 expression and survival of NSCLC patients treated with cisplatin-based chemotherapy

To investigate the relationship between ABCG4 expression and the clinical outcome of NSCLC patients treated with cisplatin-based combination chemotherapy, univariate and multivariate analyses were performed using the Cox proportional hazards model to determine whether the prognostic value of ABCG4 was independent from other factors. In univariate analysis, gender, smoking habit, age, and histology did not influence prognosis (all *P*>0.05). ABCG4 expression (hazard ratio [HR]: 2.284), high TNM stage (HR: 3.124), and poor differentiation (HR: 1.869) were significantly associated with a poor prognosis (Table 4). Moreover, factors that were associated with a *P*-value <0.05 in univariate analysis were included into a multivariate analysis. Results showed that, besides the TNM stage (HR: 3.098) and differentiation (HR: 1.811), ABCG4 expression was an independent prognostic factor for survival of patients with NSCLC. The ABCG4-positive value for OS yielded a HR of 2.236 (Table 4). These results indicated that ABCG4 expression was an adverse prognostic factor in patients with NSCLC treated with cisplatin-based chemotherapy.

**Table 4:** Cox Proportional Hazards Model Analysis of Variables Affecting Overall Survival in NSCLC Patients.

Variables	Univariate analysis		Multivariate analysis	
	HR (95% CI)	*P*	HR (95% CI)	*P*
**Gender male vs. female**	0.922(0.638–1.332)	0.665		
**Smoking (packs per year) ≥20 vs. <20**	0.893(0.618–1.289)	0.545		
**Age (years) ≥60 vs. <60**	0.979(0.676–1.420)	0.913		
**Histology squamous vs. adenocarcinoma**	1.199(0.830–1.732)	0.333		
**Differentiation poorly vs. moderately+well**	1.869(1.288–2.712)	0.001	1.811(1.239–2.647)	0.002
**TNM stage II vs. III+IV**	3.124(1.997–4.887)	<0.001	3.098(1.928–4.981)	<0.001
**ABCG4 expression** * **negative vs. positive**	2.284(1.570–3.324)	<0.001	2.236(1.505–3.321)	<0.001

*Note*: HR: hazard ratio; 95% CI: 95% confidence interval.

*: ABCG4 expression was determined by immunohistochemical analysis. Negative:-; Positive: +, ++ and +++.

**Fig 4:**
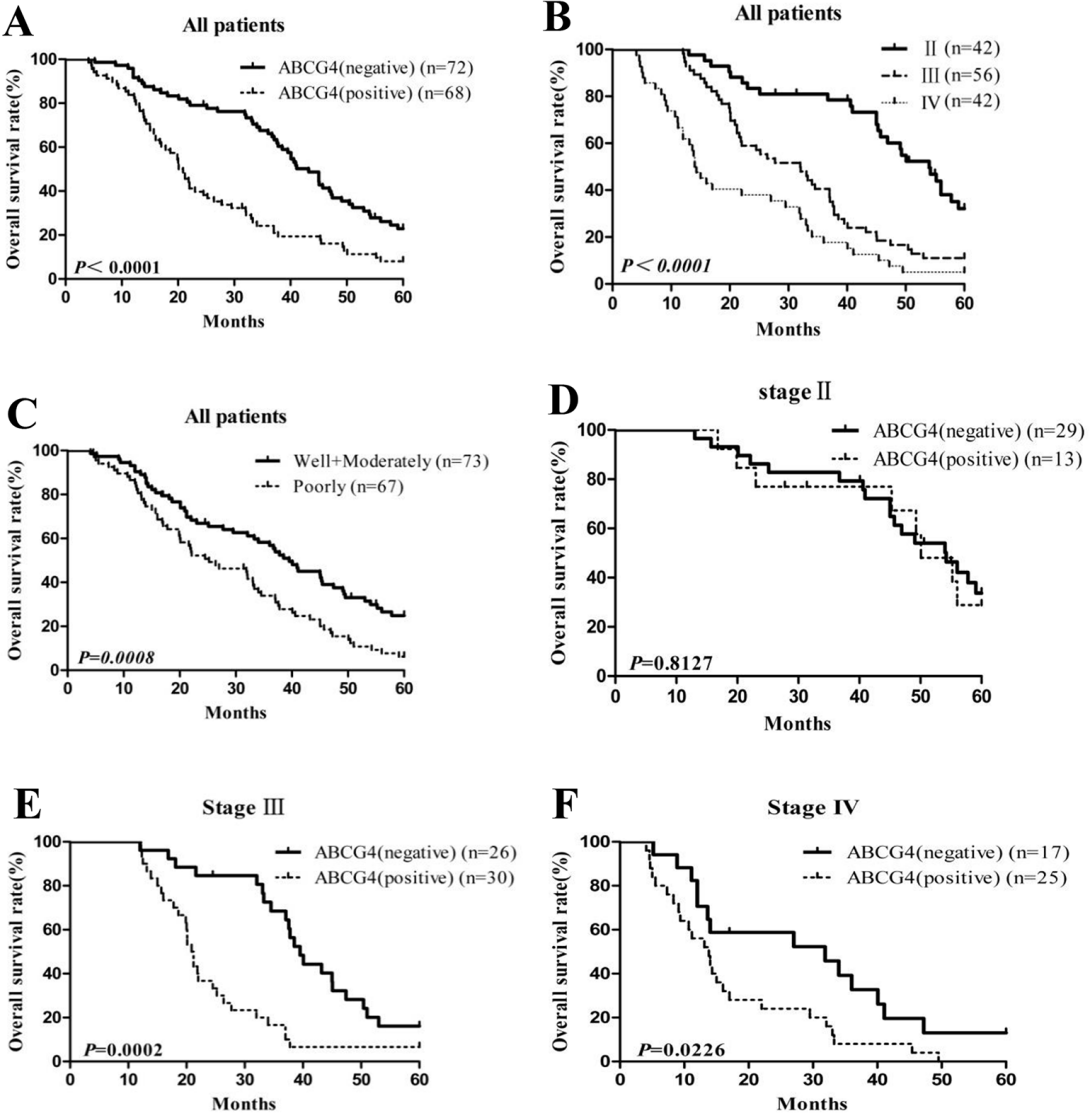
Correlation between ABCG4 expression status and prognosis of NSCLC patients treated with cisplatin-based chemotherapy. (A) Kaplan-Meier curves were plotted to determine cumulative survival rate of NSCLC patients based on ABCG4 expression (negative *vs*. positive). (B) Kaplan-Meier curves were plotted to determine cumulative survival rate of NSCLC patients based on tumor node metastasis (TNM) stage (II *vs*. III *vs*. IV). (C) Kaplan-Meier curves were plotted to determine cumulative survival rate of NSCLC patients based on differentiation (poorly *vs*. moderately/well). (D) Kaplan-Meier curves were plotted to determine cumulative survival rate of NSCLC patients with TNM stage II based on ABCG4 expression (negative *vs*. positive). (E) Kaplan-Meier curves were plotted to determine cumulative survival rate of NSCLC patients with TNM stage III based on ABCG4 expression (negative *vs*. positive). (F) Kaplan-Meier curves were plotted to determine cumulative survival rate of NSCLC patients with TNM stage IV based on ABCG4 expression (negative *vs*. positive). Negative:-; Positive: +, ++ and +++.

OS curves are shown in Fig 4, according to the ABCG4 expression status. Median survival was 43.2 and 20.1 months in patients with ABCG4-negative and ABCG4-positive NSCLC, respectively (*P*<0.0001, Fig 4A). Median survival was 54, 32, and 14.15 months in patients with stages II, III, and IV NSCLC, respectively (*P*<0.0001, Fig 4B). Median survival was 39.5 and 25.2 months in patients with well/moderately and poorly differentiated NSCLC, respectively (*P* = 0.0008, Fig 4C). Median survival in the ABCG4-negative group was 39.5 months, compared with 21.05 months in the ABCG4-positive group in stage III (*P* = 0.0002, Fig 4E). Furthermore, median survival in the ABCG4-negative group was 31.9 months, compared with 13.8 months in the ABCG4-positive group in stage IV (*P* = 0.0226, Fig 4F). However, median survival in the ABCG4-negative group was 54.2 months, compared with 50.1 months in the ABCG4-positive group in stage II, and there was no significant difference in OS with respect to ABCG4 expression (*P* = 0.8127, Fig 4D).

## Discussion

It has already been established that ABC transporters are involved in MDR and have an effect on the efficacy of chemotherapy regimens [11,12]. The purpose of this study was to investigate ABCG4 and its expression in NSCLC in order to assess if there is an association between ABCG4 expression and patients’ outcome after chemotherapy. In this study, we provided solid evidence for ABCG4 mRNA and protein expression in some samples of NSCLC tissue but not in any of the normal lung tissues. Moreover, ABCG4 expression was associated with different grades of differentiation, TNM stage, and histological type. Univariate and multivariate analyses suggested that TNM stage, differentiation, and ABCG4 expression were independent factors involved in prognosis in this population of NSCLC patients treated with cisplatin-based chemotherapy. These results suggested a worse survival in patients with ABCG4-positive NSCLC treated with cisplatin-based chemotherapy.

ABCG4 expression was significantly higher in NSCLC tissue samples compared with normal lung tissue samples. Moreover, ABCG4 expression was associated with different grades of differentiation, TNM stage, and histological type. Previous studies have shown that despite similarities in sequence, structure, and function of MDR between ABCG2 (BCRP) and ABCG4, ABCG2 is not associated with gender, smoking history, performance status (PS), histological type, and TNM stage in NSCLC [35,36], while we observed associations between ABCG4 and histological type, differentiation grade, and TNM stage. One explanation may be that the specificity and diversity of sequence and structure between ABCG subfamily transporter genes contribute to this discrepancy. In addition, expression of ABC subfamily transporter proteins vary with clinical variables; for instance, ABCB1 expression is significantly higher in females than in males, and higher in non-squamous cell carcinoma than in squamous cell carcinoma in NSCLC. However, ABCC2 expression is significantly higher in stage IV NSCLC compared with stage IIIB, while there is no significant correlation between ABCC1/ABCC3 expression and clinical variables in NSCLC [35]. This discrepancy needs to be investigated further. ABCG4 also shares some similarities with ABCG1. However, basal ATPase activity of ABCG4 is lower than that of ABCG1, and ABCG4 is not inhibited by the same drugs as ABCG1 [37]. However, the exact interaction and the role of this interaction between ABCG1 and ABCG4 still need to be elucidated [38].

Previous studies have shown that ABCG4 mediates the efflux of endogenous steroids in cells and transfers them to high-density lipoproteins (HDL), thus helping to maintain cholesterol homeostasis [30,39]. However, Castanys-Muñoz et al. have discovered that over-expression of ABCG4 is also involved in alkyl-phospholipid analogues (miltefosine, edelfosine, and perifosine) resistance in *Leishmania* [33]. The results of the present study suggest a shorter survival in patients with ABCG4-positive NSCLC treated with cisplatin-based chemotherapy. We speculate that patients with ABCG4-positive NSCLC may show resistance to cisplatin-based chemotherapy. We suggest that ABCG4 may serve as a molecular target for reducing drug resistance in NSCLC. The relationship between ABCG4 and drug resistance, and especially multi-drug resistance, will be more specifically addressed in future studies.

On the other hand, this was the first study to investigate the relationship between ABCG4 expression and prognosis in patients with NSCLC. Based on OS curves, ABCG4-positive patients had a shorter median survival time than ABCG4-negative patients except for stage II NSCLCs (*P* = 0.8127). Median survival was not significantly different in stage II NSCLCs, which may be because ABCG4 expression levels were relatively low in stage II NSCLCs, and that the number of ABCG4-positive patients with stage II NSCLC was relatively small (n = 13). Therefore, this result should be interpreted with caution. The univariate and multivariate analyses showed that besides TNM stage and differentiation, ABCG4 was independently associated with OS in NSCLCs treated with cisplatin-based combination chemotherapy. These results suggest that ABCG4 expression may be independent risk factor of prognosis and predictive biomarker in patients with NSCLC treated with cisplatin-based combination chemotherapy. Our results are consistent with those of Yoh et al. and Ota et al., which support a predictive role for ABCG2 in prognosis of NSCLC patients treated with cisplatin-based combination chemotherapy [35,36]. In addition, we speculate that ABCG4 mRNA expression may be used to design individualized chemotherapy for NSCLC patients.

This study had some limitations. Indeed, the sample size was small, and a larger sample size would be preferable. We also had no healthy control subjects because lung resection in healthy individuals is highly invasive and unethical. However, the control tissues from the patients with NSCLC were histologically different from the tumor tissues and >5 cm away from the tumors, so we presumed this was an effective control under the circumstances. Finally, intracellular localization of ABCG4 will be assessed in a future work. Further studies will be perform on different NSCLC cell lines that overexpress ABCG4 alone or in combination with other ABC transporters, which should help to elucidate the exact role and function of ABCG4 in NSCLC.

## Conclusions

This study strongly suggests that high ABCG4 expression is associated with poor prognosis in patients with NSCLC treated with cisplatin-based chemotherapy, and patients with ABCG4-positive NSCLC may show resistance to cisplatin-based chemotherapy. Moreover, the present study is the first to indicate a potential role for ABCG4 expression as an independent predictive factor of poor prognosis in NSCLC patients treated with cisplatin-based combination chemotherapy. However, further study with larger sample size and cells are needed to confirm these results, and the specific pathway and mechanism driving this effect need further study. These may lead to a novel approach for the reversal of resistance to chemotherapy drugs by understanding ABCG4 and its mechanism in NSCLC.

## Supporting Information

S1 Fig Full western blot of ABCG4 protein (60 kDa) expression. β-actin (43 kDa) was used as an internal reference.
(PDF)

S1 File Raw data for cox proportional hazards model analysis and Kaplan-Meier curves. Note: Gender: 0, male; 1, female. Event: 0, death; 1, censored data. Age: 0, ≥60; 1,<60. Smoking: 0, ≥20 packs; 1, <20 packs. Histology: 0, Adenocarcinoma; 1, Squamous cell carcinoma. Differentiation: 0, Poor; 1, Moderate/well. Stage: 0, Ⅲ+Ⅳ; 1, Ⅱ. ABCG4: 0, (+,++,+++); 1, (-).
(XLS)

## References

[1] Group NM-aC. Preoperative chemotherapy for non-small-cell lung cancer: a systematic review and meta-analysis of individual participant data. Lancet. 2014;383: 1561–1571. 10.1016/S0140-6736(13)62159-5 24576776 PMC4022989

[2] Xian-Jun F, Xiu-Guang Q, Li Z, Hui F, Wan-Ling W, Dong L, et al ERCC1 and BRCA1 mRNA expression predicts the clinical outcome of non-small cell lung cancer receiving platinum-based chemotherapy. Pak J Med Sci. 2014;30: 488–492. 10.12669/pjms.303.4187 24948964 PMC4048491

[3] Askoxylakis V, Thieke C, Pleger ST, Most P, Tanner J, Lindel K, et al Long-term survival of cancer patients compared to heart failure and stroke: a systematic review. BMC Cancer. 2010;10: 105 10.1186/1471-2407-10-105 20307299 PMC2851688

[4] NSCLC Meta-analyses Collaborative Group, Arriagada R, Auperin A, Burdett S, Higgins JP, Johnson DH, et al Adjuvant chemotherapy, with or without postoperative radiotherapy, in operable non-small-cell lung cancer: two meta-analyses of individual patient data. Lancet. 2010;375: 1267–1277. 10.1016/S0140-6736(10)60059-1 20338627 PMC2853682

[5] Network NCC. Clinical Practice Guidelines in Oncology™: Non-Small Cell Lung Cancer(v.4). 2014. http://www.nccn.org.

[6] Arrieta O, Gonzalez-De la Rosa CH, Arechaga-Ocampo E, Villanueva-Rodriguez G, Ceron-Lizarraga TL, Martínez-Barrera L, et al Randomized phase II trial of All-trans-retinoic acid with chemotherapy based on paclitaxel and cisplatin as first-line treatment in patients with advanced non-small-cell lung cancer. J Clin Oncol. 2010;28: 3463–3471. 10.1200/JCO.2009.26.6452 20547984

[7] Reungwetwattana T, Weroha SJ, Molina JR. Oncogenic pathways, molecularly targeted therapies, and highlighted clinical trials in non-small-cell lung cancer (NSCLC). Clin Lung Cancer. 2012;13: 252–266. 10.1016/j.cllc.2011.09.004 22154278

[8] Li J, Li ZN, Du YJ, Li XQ, Bao QL, Chen P. Expression of MRP1, BCRP, LRP, and ERCC1 in advanced non-small-cell lung cancer: correlation with response to chemotherapy and survival. Clin Lung Cancer. 2009;10: 414–421. 10.3816/CLC.2009.n.078 19900859

[9] Hall MD, Marshall TS, Kwit AD, Miller Jenkins LM, Dulcey AE, Madigan JP, et al Inhibition of glutathione peroxidase mediates the collateral sensitivity of multidrug-resistant cells to tiopronin. J Biol Chem. 2014;289: 21473–21489. 10.1074/jbc.M114.581702 24930045 PMC4118110

[10] Kunicka T, Soucek P. Importance of ABCC1 for cancer therapy and prognosis. Drug Metab Rev. 2014;46: 325–342. 10.3109/03602532.2014.901348 24670052

[11] Fukuda Y, Schuetz JD. ABC transporters and their role in nucleoside and nucleotide drug resistance. Biochem Pharmacol. 2012;83: 1073–1083. 10.1016/j.bcp.2011.12.042 22285911 PMC3319017

[12] Kim YH, Ishii G, Goto K, Ota S, Kubota K, Murata Y, et al Expression of breast cancer resistance protein is associated with a poor clinical outcome in patients with small-cell lung cancer. Lung Cancer. 2009;65: 105–111. 10.1016/j.lungcan.2008.10.008 19036469

[13] Hollenstein K, Dawson RJ, Locher KP. Structure and mechanism of ABC transporter proteins. Curr Opin Struct Biol. 2007;17: 412–418. 17723295 10.1016/j.sbi.2007.07.003

[14] Tamaki A, Ierano C, Szakacs G, Robey RW, Bates SE. The controversial role of ABC transporters in clinical oncology. Essays Biochem. 2011;50: 209–232. 10.1042/bse0500209 21967059 PMC6944313

[15] Silverton L, Dean M, Moitra K. Variation and evolution of the ABC transporter genes ABCB1, ABCC1, ABCG2, ABCG5 and ABCG8: implication for pharmacogenetics and disease. Drug Metabol Drug Interact. 2011;26: 169–179. 10.1515/DMDI.2011.027 22098604 PMC7372709

[16] Prochazka L, Koudelka S, Dong LF, Stursa J, Goodwin J, Neca J, et al Mitochondrial targeting overcomes ABCA1-dependent resistance of lung carcinoma to alpha-tocopheryl succinate. Apoptosis. 2013;18: 286–299. 10.1007/s10495-012-0795-1 23299931

[17] Szakacs G, Varadi A, Ozvegy-Laczka C, Sarkadi B. The role of ABC transporters in drug absorption, distribution, metabolism, excretion and toxicity (ADME-Tox). Drug Discov Today. 2008;13: 379–393. 10.1016/j.drudis.2007.12.010 18468555

[18] Falasca M, Linton KJ. Investigational ABC transporter inhibitors. Expert Opin Investig Drugs. 2012;21: 657–666. 10.1517/13543784.2012.679339 22493979

[19] Ross DD, Yang W, Abruzzo LV, Dalton WS, Schneider E, Lage H, et al Atypical multidrug resistance: breast cancer resistance protein messenger RNA expression in mitoxantrone-selected cell lines. J Natl Cancer Inst. 1999;91: 429–433. 10070941 10.1093/jnci/91.5.429

[20] Pohl A, Devaux PF, Herrmann A. Function of prokaryotic and eukaryotic ABC proteins in lipid transport. Biochim Biophys Acta. 2005;1733: 29–52. 15749056 10.1016/j.bbalip.2004.12.007

[21] van Meer G, Halter D, Sprong H, Somerharju P, Egmond MR. ABC lipid transporters: extruders, flippases, or flopless activators? FEBS Lett. 2006;580: 1171–1177. 16376334 10.1016/j.febslet.2005.12.019

[22] Graf GA, Yu L, Li WP, Gerard R, Tuma PL, Cohen JC, et al ABCG5 and ABCG8 are obligate heterodimers for protein trafficking and biliary cholesterol excretion. J Biol Chem. 2003;278: 48275–48282. 14504269 10.1074/jbc.M310223200

[23] Langheim S, Yu L, von Bergmann K, Lutjohann D, Xu F, Hobbs HH, et al ABCG5 and ABCG8 require MDR2 for secretion of cholesterol into bile. J Lipid Res. 2005; 46: 1732–1738. 15930516 10.1194/jlr.M500115-JLR200

[24] de Jonge-Peeters SD, Kuipers F, de Vries EG, Vellenga E. ABC transporter expression in hematopoietic stem cells and the role in AML drug resistance. Crit Rev Oncol Hematol. 2007;62: 214–226. 17368038 10.1016/j.critrevonc.2007.02.003

[25] Murphy AJ, Bijl N, Yvan-Charvet L, Welch CB, Bhagwat N, Reheman A, et al Cholesterol efflux in megakaryocyte progenitors suppresses platelet production and thrombocytosis. Nat Med. 2013;19: 586–594. 10.1038/nm.3150 23584088 PMC3683965

[26] Ruiz JL, Fernandes LR, Levy D, Bydlowski SP. Interrelationship between ATP-binding cassette transporters and oxysterols. Biochem Pharmacol. 2013;86: 80–88. 10.1016/j.bcp.2013.02.033 23500544

[27] Annilo T, Tammur J, Hutchinson A, Rzhetsky A, Dean M, Allikmets R. Human and mouse orthologs of a new ATP-binding cassette gene, ABCG4. Cytogenet Cell Genet. 2001;94: 196–201. 11856881 10.1159/000048816

[28] Oldfield S, Lowry C, Ruddick J, Lightman S. ABCG4: a novel human white family ABC-transporter expressed in the brain and eye. Biochim Biophys Acta. 2002;1591: 175–179. 12183068 10.1016/s0167-4889(02)00269-0

[29] Koshiba S, Ito T, Shiota A, Wakabayashi K, Ueda M, Ichinose H, et al Development of polyclonal antibodies specific to ATP-binding cassette transporters human ABCG4 and mouse Abcg4: site-specific expression of mouse Abcg4 in brain. J Exp Ther Oncol. 2007;6: 321–333. 18038765

[30] Fujiyoshi M, Ohtsuki S, Hori S, Tachikawa M, Terasaki T. 24S-hydroxycholesterol induces cholesterol release from choroid plexus epithelial cells in an apical- and apoE isoform-dependent manner concomitantly with the induction of ABCA1 and ABCG1 expression. J Neurochem. 2007;100: 968–978. 17101031 10.1111/j.1471-4159.2006.04240.x

[31] Klucken J, Buchler C, Orso E, Kaminski WE, Porsch-Ozcurumez M, Liebisch G, et al ABCG1 (ABC8), the human homolog of the Drosophila white gene, is a regulator of macrophage cholesterol and phospholipid transport. Proc Natl Acad Sci U S A. 2000;97: 817–822. 10639163 10.1073/pnas.97.2.817 PMC15414

[32] Wang N, Lan D, Chen W, Matsuura F, Tall AR. ATP-binding cassette transporters G1 and G4 mediate cellular cholesterol efflux to high-density lipoproteins. Proc Natl Acad Sci U S A. 2004;101: 9774–9779. 15210959 10.1073/pnas.0403506101 PMC470750

[33] Castanys-Munoz E, Alder-Baerens N, Pomorski T, Gamarro F, Castanys S. A novel ATP-binding cassette transporter from Leishmania is involved in transport of phosphatidylcholine analogues and resistance to alkyl-phospholipids. Mol Microbiol. 2007;64: 1141–1153. 17542911 10.1111/j.1365-2958.2007.05653.x

[34] Zhao J, Zhou Y, Zhang Z, Tian F, Ma N, Liu T, et al (2010) Upregulated fascin1 in non-small cell lung cancer promotes the migration and invasiveness, but not proliferation. Cancer Lett. 2010;290: 238–247. 10.1016/j.canlet.2009.09.013 19819618

[35] Yoh K, Ishii G, Yokose T, Minegishi Y, Tsuta K, Goto K, et al Breast cancer resistance protein impacts clinical outcome in platinum-based chemotherapy for advanced non-small cell lung cancer. Clin Cancer Res. 2004;10: 1691–1697. 15014021 10.1158/1078-0432.ccr-0937-3

[36] Ota S, Ishii G, Goto K, Kubota K, Kim YH, Kojika M, et al Immunohistochemical expression of BCRP and ERCC1 in biopsy specimen predicts survival in advanced non-small-cell lung cancer treated with cisplatin-based chemotherapy. Lung Cancer. 2009;64: 98–104. 10.1016/j.lungcan.2008.07.014 18823676

[37] Cserepes J, Szentpetery Z, Seres L, Ozvegy-Laczka C, Langmann T, Schmitz G, et al Functional expression and characterization of the human ABCG1 and ABCG4 proteins: indications for heterodimerization. Biochem Biophys Res Commun. 2004;320: 860–867. 15240127 10.1016/j.bbrc.2004.06.037

[38] Kerr ID, Haider AJ, Gelissen IC. The ABCG family of membrane-associated transporters: you don't have to be big to be mighty. Br J Pharmacol. 2011;164: 1767–1779. 10.1111/j.1476-5381.2010.01177.x 21175590 PMC3246702

[39] Tarr PT, Edwards PA. ABCG1 and ABCG4 are coexpressed in neurons and astrocytes of the CNS and regulate cholesterol homeostasis through SREBP-2. J Lipid Res. 2008;49: 169–182. 17916878 10.1194/jlr.M700364-JLR200

