# Supplementary figures and images for: High ABCG4 Expression Is Associated with Poor Prognosis in Non-Small-Cell Lung Cancer Patients Treated with Cisplatin-Based Chemotherapy
Source: PLoS One. 2015 Aug 13;10(8):e0135576. doi: 10.1371/journal.pone.0135576 (PMC4535915; doi:10.1371/journal.pone.0135576)

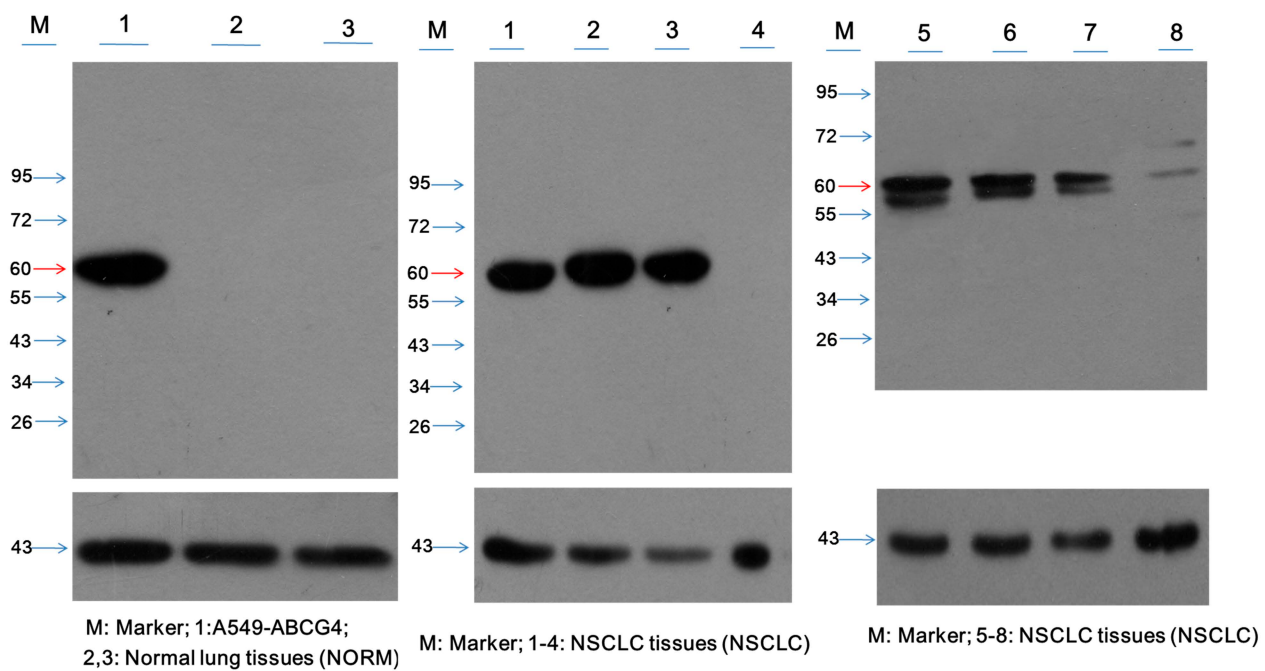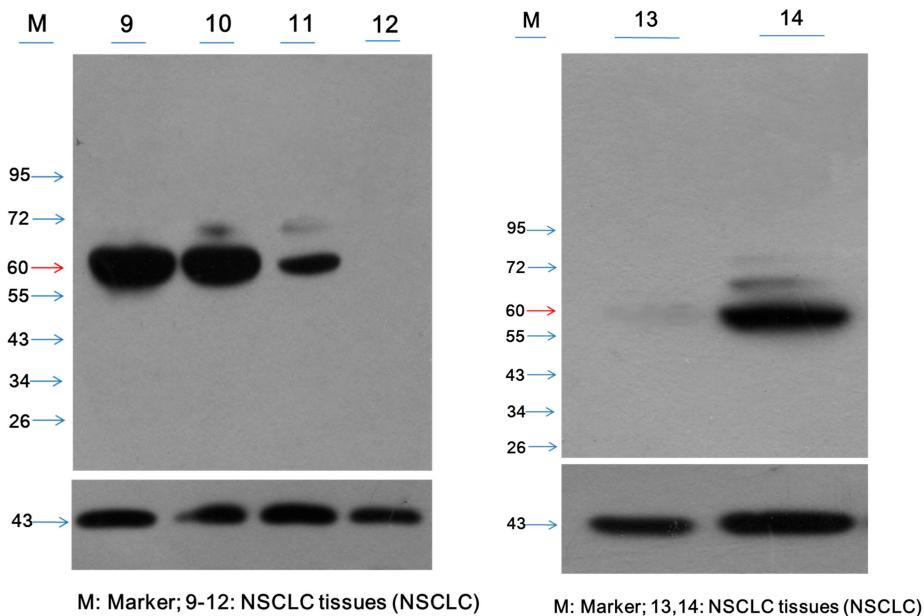

Supplement: S1 Fig — β-actin (43 kDa) was used as an internal reference. (PDF) [file pone.0135576.s001.pdf]
